# MicroRNA‐195 suppresses the progression of lung adenocarcinoma by directly targeting apelin

**DOI:** 10.1111/1759-7714.13087

**Published:** 2019-05-09

**Authors:** Yongchun Zhou, Ming Zhao, Yaxi Du, Yajie Liu, Guangqiang Zhao, Lianhua Ye, Quan Li, Hongsheng Li, Xiaoxiong Wang, Xin Liu, Yinjin Guo, Junxi Liu, Yunchao Huang

**Affiliations:** ^1^ The International Cooperation Key Laboratory of Regional Tumor in High Altitude Area Molecular Diagnostic Center, The Third Affiliated Hospital of Kunming Medical University Kunming China; ^2^ Department of Medical Records The Third Affiliated Hospital of Kunming Medical University Kunming China; ^3^ Kunming Medical University Key Laboratory of Lung Cancer Research of Yunnan Province and Kunming City Kunming China; ^4^ Department of Asset Management The Third Affiliated Hospital of Kunming Medical University Kunming China; ^5^ Department of Cardiothoracic Surgery The Third Affiliated Hospital of Kunming Medical University Kunming China; ^6^ Cancer Research Laboratory, The Cancer Research Institute The Third Affiliated Hospital of Kunming Medical University Kunming China

**Keywords:** Apelin, lung adenocarcinoma, microRNA‐195, migration, proliferation

## Abstract

**Background:**

Apelin plays an important role in many types of tumors. We aimed to identify the effects of miR‐195 on inhibiting apelin and clarify the regulating mechanism of miR‐195‐apelin in lung adenocarcinoma cells.

**Methods:**

We detected the expression levels of apelin and miR‐195 in lung adenocarcinoma tissues and lung cancer cell lines using Western blotting and quantitative reverse transcription PCR assay, respectively. Luciferase reporter assay was used to confirm the target gene of miR‐195. The effects of miR‐195 and apelin on the proliferation and cell cycle of lung adenocarcinoma cells were assessed by methyl thiazolyl tetrazolium and colony formation assays, and flow cytometry. Wound‐healing and transwell invasion experiments were employed to examine cellular migration and invasion. A tumor xenograft model was then used to investigate the role of miR‐195 on tumor growth in vivo.

**Results:**

The expression level of apelin and miR‐195 showed an inverse correlation in lung adenocarcinoma tissues and cell lines. Luciferase reporter assay suggested that miR‐195 directly targets apelin messenger RNA. Overexpression of miR‐195 significantly inhibited the proliferation, migration, and invasion of lung adenocarcinoma cells in vitro and suppressed tumor growth in vivo. Further analysis revealed that *apelin* is one of the functional target genes of miR‐195, and the overexpression of apelin efficiently inhibits the promotion of cell proliferation and invasion mediated by miR‐195 mimics in lung adenocarcinoma cells.

**Conclusions:**

Our data constitute evidence that miR‐195 inhibits lung adenocarcinoma cell proliferation and invasion though targeting apelin and provides novel insight into the mechanism underlying the development of lung adenocarcinoma.

## Introduction

Lung adenocarcinoma is the most common histological subtype of non‐small cell lung cancer (NSCLC), and is a significant threat to public health worldwide.^1^ Lung adenocarcinoma is associated with high mortality, with a five‐year overall survival (OS) rate of < 20%, because of diagnosis at an advanced stage and early metastasis, which is not the optimal period for treatment.^2^ Despite recent advances in early diagnostic methods and improvements in chemotherapy, radiation, and surgical treatments, the prognosis of patients with lung adenocarcinoma remains very poor.^3^ Therefore, a better understanding of the molecular mechanisms underlying lung adenocarcinoma development will be useful to identify valid diagnostic biomarkers and more effective therapeutic strategies.

The *APLN* gene encodes the peptide known as apelin, which was initially isolated and purified from bovine stomach extracts and has been identified as an endogenous ligand for the human orphan G protein‐coupled receptor APJ. In addition, the interaction of the APJ receptor and ligand plays a role via coupling with the inhibitory G protein (G_i_).^4^ The *APLN* gene encodes 77 amino acid pre‐proproteins that, following enzymatic cleavage, produce four different peptide fragments: apelin‐36, apelin‐17, apelin‐13, and apelin‐12 from the C‐terminus.^5^ It has been reported that apelin, as a novel angiogenic factor, is involved in the regulation of cardiovascular function and promotion of angiogenesis, lymphangiogenesis, and tumor growth in vivo, and the angiogenic potential of apelin is similar to that of VEGF.^6^, ^7^ Previous studies demonstrated that apelin has aberrantly high expression and high apelin protein levels are associated with cell proliferation, angiogenesis, and prognosis in various cancers, including breast, lung, oral, liver, and colon cancers.^8^, ^9^, ^10^, ^11^, ^12^ However, the molecular mechanisms involved in the upstream regulation of apelin expression in lung adenocarcinoma remain unknown.

MicroRNAs (miRNAs) are a class of endogenous non‐coding RNAs (18–22 nucleotides) that negatively regulate gene expression at the posttranscriptional level through specific binding to the 3′‐untranslated regions (UTRs) of target messenger RNAs (mRNAs).^13^, ^14^ An accumulating body of evidence has revealed that the deregulation of miRNA expression may lead to various diseases, including NSCLC.^15^, ^16^, ^17^ Recently, it has been reported that miR‐195 is significantly downregulated in NSCLC tumor tissues and overexpression of miR‐195 inhibits cell proliferation, migration, and invasion in NSCLC cell lines via targeting HDGF, IGF1R1, CHEK1, CCND3, and BIRC5.^18^, ^19^, ^20^, ^21^ However, the specific mechanisms involved in miR‐195 inhibition of the progression of lung adenocarcinoma have not been completely expounded.

In the present study, we found that apelin exhibited a higher level of expression, whereas miR‐195 was significantly downregulated in human lung adenocarcinoma samples and cell lines, in which apelin mRNA expression was inversely correlated with the miR‐195 level. Both high levels of apelin and low levels of miR‐195 in lung adenocarcinoma tissues are associated with poor survival. We also determined the effects of miR‐195 on the proliferation, migration, and invasion of lung adenocarcinoma cells in vitro and in vivo. We confirmed that miR‐195 exerts a tumor suppressor role by targeting apelin mRNA in lung adenocarcinoma cells. Our results show that miR‐195 functions as a tumor suppressor in lung adenocarcinoma and implicate miR‐195‐apelin as a potential diagnostic and therapeutic target for human lung adenocarcinoma.

## Methods

### Tissue samples

Human lung adenocarcinoma and adjacent healthy lung tissues (> 5 cm away from tumor tissues) were obtained from the Third Affiliated Hospital of Kunming Medical University between June 2012 and December 2016 with the patients’ informed consent. The category of lung adenocarcinoma tissues samples was confirmed by pathological analysis. All of the tissue samples were immediately snap‐frozen in liquid nitrogen and stored at −80°C until use. None of the patients received any treatment, such as radiotherapy or chemotherapy, prior to surgery. The clinical characteristics of the patients included are summarized in Table 1.

**Table 1:** Clinical characteristics of patients with lung adenocarcinoma (*n* = 50) and correlations with apelin expression

		Apelin expression level		
Variable	Total	Low	High	*P*
Total, N	50			
Median age, years (*n* ± SD)	59.0 ± 6.85			
Age range, years	40–75			
Gender, N (%)				0.544
Male	28 (56.0)	13	15	
Female	22 (44.0)	10	12	
Smoking history, N (%)				0.165
Yes	30 (60.0)	12	18	
No	20 (40.0)	11	9	
Histological grade, N (%)				0.767
Poorly differentiated	27 (54.0)	10	17	
Well/moderately differentiated	23 (46.0)	13	10	
TNM stage, N (%)				0.011^a^
I + II	32 (64.0)	22	10	
III + IV	18 (36.0)	1	17	
Lymph node metastasis, N (%)				0.007^a^
Yes	21 (42.0)	2	19	
No	29 (58.0)	21	8	

a * Significant difference (*P* < 0.05). SD, standard deviation; TNM, tumor node metastasis.

Twenty‐eight men and 22 women were included, with a mean age at diagnosis of 59 (range: 40–75) years. The Clinical Research Ethics Committee of Kunming Medical University (Kunming, Yunnan, China), approved the study.

### Immunohistochemistry staining

Immunohistochemistry staining was used to detect APLN protein expression in lung adenocarcinoma tissues via an ultraView Universal DAB Detection Kit (Ventana, Tucson, AZ, USA) according to the manufacturer's protocol. Each tissue sample was sliced, deparaffinized, and dehydrated for subsequent antigen retrieval. The slide was first stained with the apelin antibody, which was a primary antibody, and then incubated with a secondary antibody conjugated with horseradish peroxidase (HRP) for one hour at room temperature. After the formation of the antigen‐antibody‐antibody complex, a substrate of the peroxidase and diaminobenzidine was added as chromogen. All of the slides were counterstained with hematoxylin. Two pathologists who were blinded to the patients’ prognosis and clinicopathological outcomes independently evaluated the immunostaining results. All of the images were obtained using an Olympus microscope equipped with a digital color camera and software.

### Cell culture and transfection

Four lung cancer cell lines (A549, GLC‐82, XWLC‐05, and YTMLC) and the normal human lung epithelial cell line BEAS‐2B were purchased from Shanghai Ao Lu Biotechnology Co. Ltd. (Shanghai, China). All of the lung cancer cells were cultured in RPMI 1640 medium (Gibco, Thermo Fisher Scientific, Waltham, MA, USA) supplemented with 10% fetal bovine serum (FBS, Gibco) and 100 IU/mL of penicillin. The BEAS‐2B cells were cultured in Dulbecco's modified Eagle's medium (DMEM) with 10% FBS and 100 IU/mL of penicillin. The cultures were maintained in a humidified atmosphere at 37°C with 5% CO_2_ and the media were changed every three days.

MiR‐195 mimics and negative control (NC) were purchased from RiboBio (Guangzhou, China). Apelin‐specific small interfering RNAs (siRNAs, si‐apelin) and control siRNAs (si‐NC) were purchased from GeneChem (Shanghai, China). The cells were cultured to 70~80% confluence, followed by transfection with miR‐195 mimics (50 nM) or NC using Lipofectamine 2000 Reagent (Invitrogen, Waltham, MA, USA) according to the manufacturer's protocol. The expression vector of red fluorescent protein (RFP) was used to monitor the transfection efficiency. The cells were cultured in transfection media for six hours, and then the media were replaced by complete medium for the subsequent assays.

### RNA extraction and quantitative reverse transcription PCR

Total RNA was extracted from lung cancer tissues and cells using TRIzol reagent (Invitrogen) according to the manufacturer's instructions. Complementary DNA (cDNA) was then synthesized using a Reverse Transcriptase M‐MLV kit with random primers (TaKaRa, Dalian, China). U6 and β‐actin were used as internal controls to normalize the quantity of cDNA used to analyze miR‐195 and apelin mRNA expression, respectively. All of the quantitative reverse transcription quantitative (qRT) PCR procedures were performed on an ABI 7500 Thermocycler (Thermo Fisher Scientific, Waltham, MA, USA). The relative miRNA and mRNA expression was quantified using the 2^−ΔΔCt^ method. The primers were as follows: apelin forward 5′‐CTGGCAGGGAGGTCGGAGGAAATT‐3′ and reverse 5′‐TGGCTACAGCAGGTGCGAGGTGAG‐3′ and β‐actin forward 5′‐AGT GTG ACG TGG ACA TCC GCA AAG‐3′ and reverse 5′‐ATC CAC ATC TGC TGG AAG GTG GAC‐3′. The specific primers for miR‐195 (HmiRQP0376) and U6 (HmiRQP9001) were purchased from RiboBio.

### Luciferase reporter assay

The apelin 3′‐UTR containing wild‐type or mutant miR‐195 seed sequence fragments was cloned into pcDNA3/EGFP at *BamHI* and *EcoRI* sites downstream of the EGFP coding region. A549 and GLC‐82 cells were seeded into 24‐well plates and then co‐transfected with luciferase reporter plasmids and miR‐195 mimics or NC using Lipofectamine 2000 Reagent (Invitrogen) when the cells were 70~80% confluent. The vector pDsRed2‐N1 (Clontech, Mountain View, CA, USA) expressing RFP was spiked in and used for normalization. After 48 hours, luciferase activities and RFP intensities were detected with an F‐4500 Fluorescence Spectrophotometer (Hitachi, Tokyo, Japan).

### Western blotting

The cells or tissues were lysed using radioimmunoprecipitation assay buffer (Solarbio, Beijing, China). The lysis solution was collected after centrifugation at 12 000 × g for 10 minutes at 4°C. The protein concentration was determined using bicinchoninic acid assay. Total protein (20 μg) was subjected to 12% sodium dodecyl sulfate polyacrylamide gel electrophoresis and then transferred onto a nitrocellulose membrane (Millipore, Bedford, MA, USA). The membrane was blocked with 5% skimmed milk and then incubated with a primary antibody overnight at 4°C. After washing, the membranes were incubated with HRP‐conjugated secondary antibody according to the manufacturer's protocol. The antibodies used in this study were as follows: rabbit anti‐apelin (1:2000, Cat. No. ab59469), rabbit anti‐glyceraldehyde 3‐phosphate dehydrogenase (GAPDH, 1:3000, Cat. No. ab9485), and goat anti‐rabbit immunoglobulin G (HRP, 1:5000, Cat. No. ab6721; Abcam, Cambridge, UK). An ECL detection system (NEL105001EA, PerkinElmer, Waltham, MA, USA) was used to visualize the specific bands.

### Methyl thiazolyl tetrazolium assay

Methyl thiazolyl tetrazolium (MTT) assay was performed to determine cell viability. Briefly, the cells were seeded at 5 × 10^3^/well in 96‐well plates and incubated overnight. The cells were transfected with miR‐195 mimics or NC and then added to 10 μL MTT solution (10 mg mL) after transfection for 24, 48, and 72 hours, respectively. After four hours of incubation the media were discarded, 150 μL dimethyl sulfoxide was added to each well, and the absorbance was detected at a 570 nm wavelength. Five replicates were performed for each group and the process was repeated three times.

### Colony formation assay

The transfected A549 and GLC‐82 cells were harvested and seeded at a density of 200 cells/well in 12‐well plates and incubated at 37°C and 5% CO_2_ in a humidified incubator for two weeks. During colony growth, the culture media were replaced every three days. Pictures were taken and the number of colonies was counted using ImageJ software (ImageJ V1.8.0). Three replicates were performed for each group and the process was repeated three times.

### Cell cycle analysis by flow cytometry

The cultured cells were seeded into the six‐well plates at a density of 1 × 10^6^ cells/well and incubated overnight. After 48 hours of transfection, the cells were collected by trypsinization, washed twice with cold phosphate buffered saline (PBS), and fixed in 70% ethanol overnight at 4°C. The fixed cells were then washed in cold PBS and resuspended in Cell Cycle Reagent (Beckman Coulter Inc., Brea, CA, USA) at 5 × 10^5^ cells mL. The cells were incubated in the dark for 15 minutes at room temperature. The cell solutions were analyzed with a flow cytometer (Beckman Coulter Inc.) to detect cell populations at different cell cycle phases.

### Cell migration and invasion assay

A wound‐healing assay was used to measure the cell migration in the six‐well plates. A fine line was scraped with a 10 μL tip in each well after the cultured cells became fully confluent. After scratching, the cells were continuously cultured in the media with 3% FBS for 72 hours. Microscopic images of the cultures were taken at 0 and 72 hours, and then wound closure was assessed using Scion Image software.

Matrigel invasion chambers (Millipore) were used to measure cell invasion ability. Briefly, 1 × 10^5^ cells were seeded in the upper chamber with media containing 0.1% FBS, while the lower chamber was filled with media with 10% FBS. After 48 hours of incubation, the non‐invading cells were removed with cotton swabs. The cells that invaded through the Matrigel into the lower chamber of the insert and then migrated through the membrane were fixed with 75% alcohol for 30 minutes, stained with crystal violet and imaged, and counted under a microscope (Nikon, Tokyo, Japan).

### Xenograft tumor study in vivo

Five week old BALB/c nude male mice weighing 20–25 g were purchased from Beijing Huafukang Bioscience Co. Inc. (Beijing, China) and used for the in vivo tumor assay. A549 cells (2 × 10^6^) that stably transfected the miR‐195 mimics or NC were suspended in 100 μL serum‐free DMEM/Matrigel (1:1). The cells were then injected subcutaneously into the flank of each mouse. Tumor volumes were measured weekly and calculated using the formula: volume (mm^3^) = 1/2 (length × width^2^). The mice were sacrificed by cervical dislocation under anesthesia with diethyl ether after 30 days. The tumor tissues were harvested, weighed, and used to determine miR‐195 and apelin expression. The Animal Ethics Committee of Kunming Medical University (Kunming, Yunnan, China) approved all animal experiments.

### Statistical analysis

All of the statistical analyses were carried out using SPSS version 20.0. The data are presented as the median ± standard deviation (SD). Differences between groups were analyzed using Student's *t*‐test and analysis of variance. The correlation between miR‐195 and apelin expression was assessed using Pearson's correlation coefficient. Kaplan–Meier survival curves were compared between the patients with high and low expression of biomarkers using the log‐rank test. *P* < 0.05 was considered statistically significant.

## Results

### Apelin is upregulated in lung adenocarcinoma and significantly correlated with patient survival

**Figure 1:**
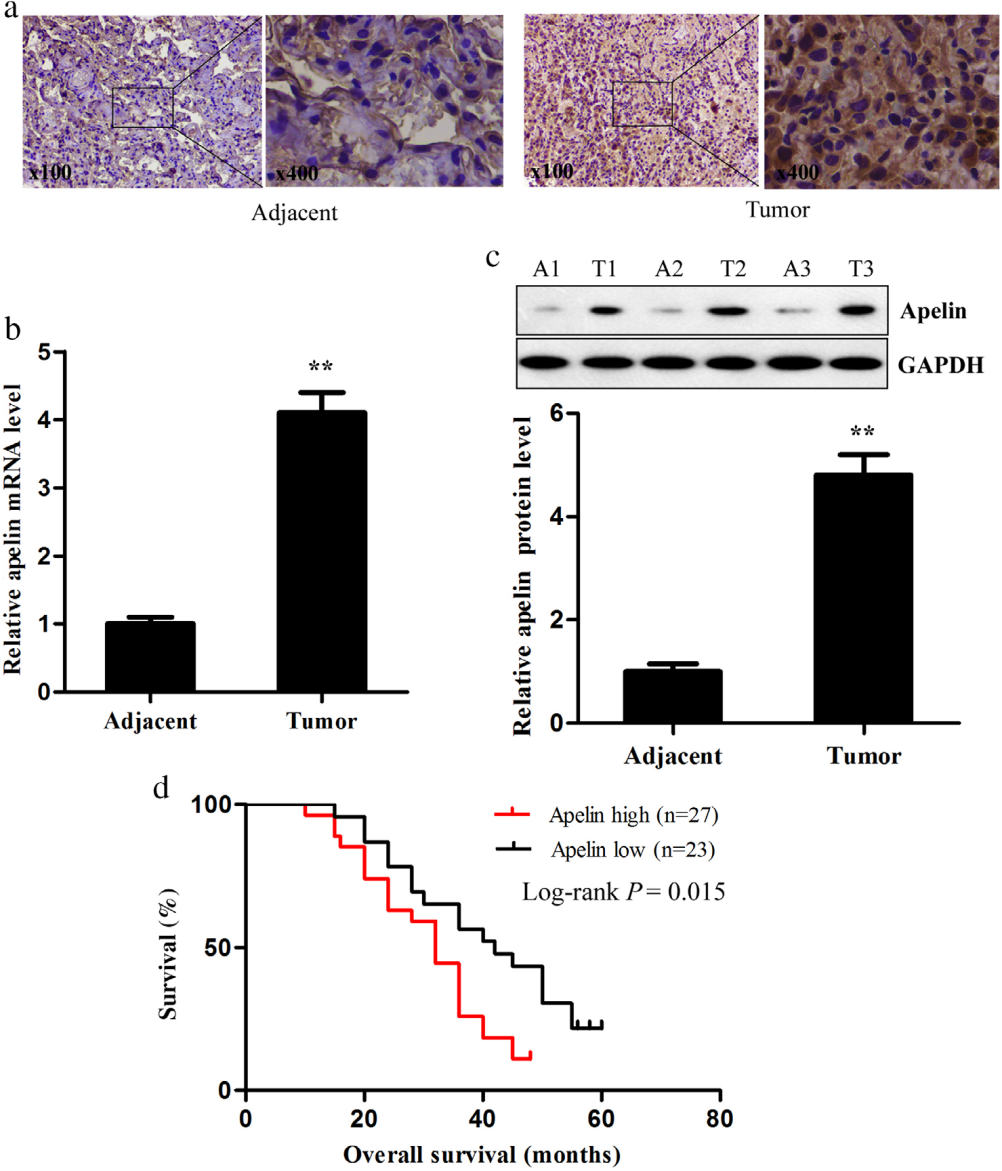
Apelin expression in lung adenocarcinoma and adjacent healthy lung tissues. (**a**) Immunohistochemical staining, (**b**) quantitative reverse transcription PCR, and (**c**) Western blotting were performed to detect apelin messenger RNA (mRNA) and protein levels in human lung adenocarcinoma and adjacent healthy lung tissues (left: × 100, right: × 400). (**d**) Kaplan–Meier analysis was performed to analyze the overall survival of lung adenocarcinoma patients with high and low expression levels of apelin (*n* = 48). ^**^ *P* < 0.01. GAPDH, glyceraldehyde 3‐phosphate dehydrogenase.

In the present study, we detected the expression of apelin in 36 paired human lung adenocarcinoma and adjacent healthy lung tissues using immunohistochemistry staining. High cytoplasmic immunoreactivity of apelin was present in the tumor cells but not in the surrounding stroma (Fig 1a). Meanwhile, using qRT‐PCR and Western blotting assay, we found that both the expression levels of apelin mRNA and protein were significantly increased in the lung adenocarcinoma tissues compared to the adjacent healthy lung tissues (Fig 1b,c). The association between apelin expression and clinical characteristics is shown in Table 1, indicating that patients with higher apelin expression had a higher tumor node metastasis (TNM) stage and a higher rate of lymph node metastasis compared to those with lower apelin levels. Furthermore, survival analysis showed that apelin expression in the 36 tumor samples was observably associated with the OS of lung adenocarcinoma patients. Patients with low apelin expression had better OS compared to those with high expression (Fig 1d). These results imply that apelin may have a tumor promoting effect in human lung adenocarcinoma.

### MiR‐195 is downregulated in lung adenocarcinoma tissues and cell lines and negatively correlated with poor prognosis and apelin expression

**Figure 2:**
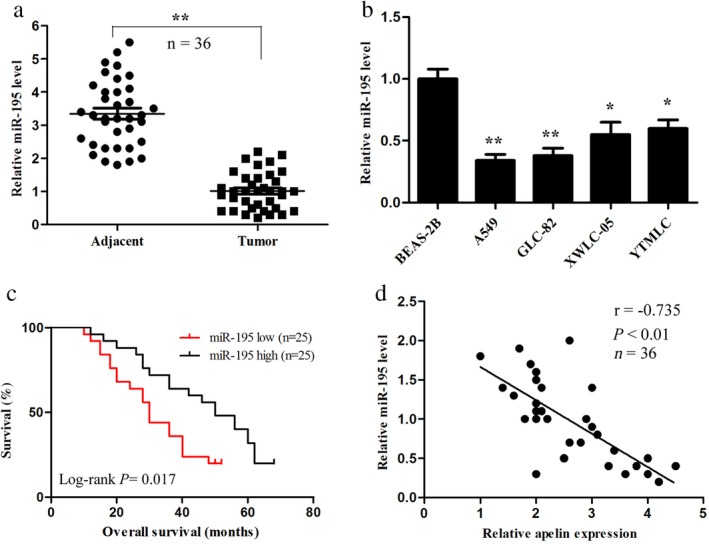
MiR‐195 expression in lung adenocarcinoma tissues and cell lines and its correlation with apelin and prognosis. (**a**) The miR‐195 level was detected by quantitative reverse transcription(qRT) PCR in human lung adenocarcinoma and adjacent healthy lung tissues. (**b**) The miR‐195 level in lung adenocarcinoma cell lines (A549, GLC‐82, XWLC‐05, and YTMLC) and normal human lung epithelial cell line BEAS‐2B was evaluated by qRT‐PCR. (**c**) Kaplan–Meier analysis was performed to analyze the overall survival of lung adenocarcinoma patients with high and low expression levels of miR‐195 (*n* = 48). (**d**) Correlation of miR‐195 levels with apelin messenger RNA levels was examined by qRT‐PCR analysis in clinical lung adenocarcinoma tissues (Pearson's correlation coefficient, r = −0.735; *n* = 36; *P* < 0.01). ^*^ *P* < 0.05, ^**^ *P* < 0.01.

We detected the expression levels of miR‐195 in the lung adenocarcinoma and adjacent healthy lung tissues using qRT‐PCR assay. MiR‐195 expression in the lung adenocarcinoma tissues was significantly lower than in the corresponding healthy lung tissues (Fig 2a). We also examined the expression levels of miR‐195 in four lung cancer cell lines (A549, GLC‐82, XWLC‐05, and YTMLC) and the normal human lung epithelial cell line BEAS‐2B. The relative expression levels of miR‐195 in these lung cancer cells were all significantly lower than in the BEAS‐2B cells (Fig 2b). These results were consistent with those of Liu *et al.* ^20^ Survival analysis showed that miR‐195 expression in the 36 tumor samples was obviously associated with the OS of lung adenocarcinoma patients and the lower expression of miR‐195 was associated with a poor prognosis (Fig 2c). In addition, Pearson's correlation analysis was performed to identify the relationship between miR‐195 and apelin mRNA expression. The expression level of miR‐195 was inversely correlated with the expression level of apelin mRNA in the lung adenocarcinoma tissues (Fig 2d). Taken together, these results suggest that decreased miR‐195 may be associated with lung adenocarcinoma.

### Apelin is a direct target of miR‐195

**Figure 3:**
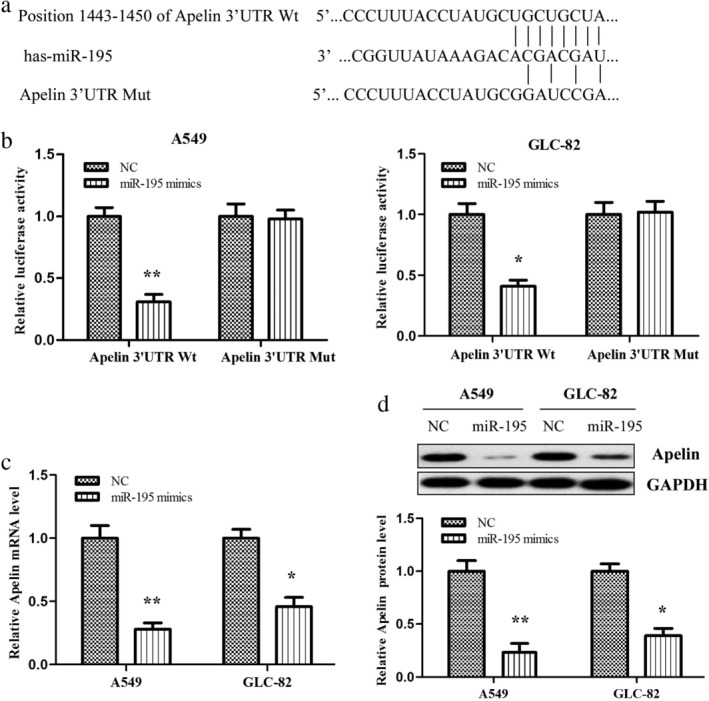
Apelin is a direct target of miR‐195. (**a**) The suspected binding of mature human miR‐195 with the wild‐type (Wt) 3′‐untranslated (UTR) region of apelin messenger RNA (mRNA) and a mutated (Mut) 3′‐UTR of apelin are shown. (**b**) A dual‐luciferase reporter assay was performed with A549 and GLC‐82 cells cotransfected with firefly luciferase constructs containing Wt 3′‐UTR region of apelin (apelin‐Wt) or the Mut 3′‐UTR region of apelin (apelin‐Mut) and miR‐195 mimics or negative control (NC). The relative luciferase activities were evaluated 48 hours after transfection. The apelin (**c**) mRNA and (**d**) protein levels in miR‐195 mimics transfected A549 and GLC‐82 cells were measured by quantitative reverse transcription PCR and Western blotting, respectively. ^*^ *P* < 0.05, ^**^ *P* < 0.01. GAPDH, glyceraldehyde 3‐phosphate dehydrogenase.

To clarify the molecular mechanism underlying apelin exerting its effects on lung adenocarcinoma cells, we identified a putative miR‐195 binding site located in the 3′‐UTR of the apelin mRNA using Pictar, TargetScan, and miRanda online tools (Fig 3a). We speculated that apelin was a potential target of miR‐195. Moreover, we validated whether miR‐195 could directly target the 3′‐UTR of apelin using the luciferase reporter assay. Our data revealed that miR‐195 significantly inhibited the firefly luciferase activity of the vector with the wild‐type 3′‐UTR of apelin; however, there was no significant effect on the vector with the mutated 3′‐UTR of apelin in A549 and GLC‐82 lung adenocarcinoma cells (Fig 3b). These results suggest that miR‐195 may directly bind to the 3′‐UTR of apelin. We also examined the effects of miR‐195 on apelin mRNA and protein expression using qRT‐PCR and Western blotting, respectively, and found that the apelin mRNA and protein levels in the miR‐195 mimics treated with A549 and GLC‐82 cells were lower than in the control cells (Fig 3c,d). These data suggest that miR‐195 can directly inhibit apelin expression by binding to its 3′‐UTR in lung adenocarcinoma cells.

### Overexpression of miR‐195 inhibits the proliferation of lung adenocarcinoma cells in vitro and in vivo

**Figure 4:**
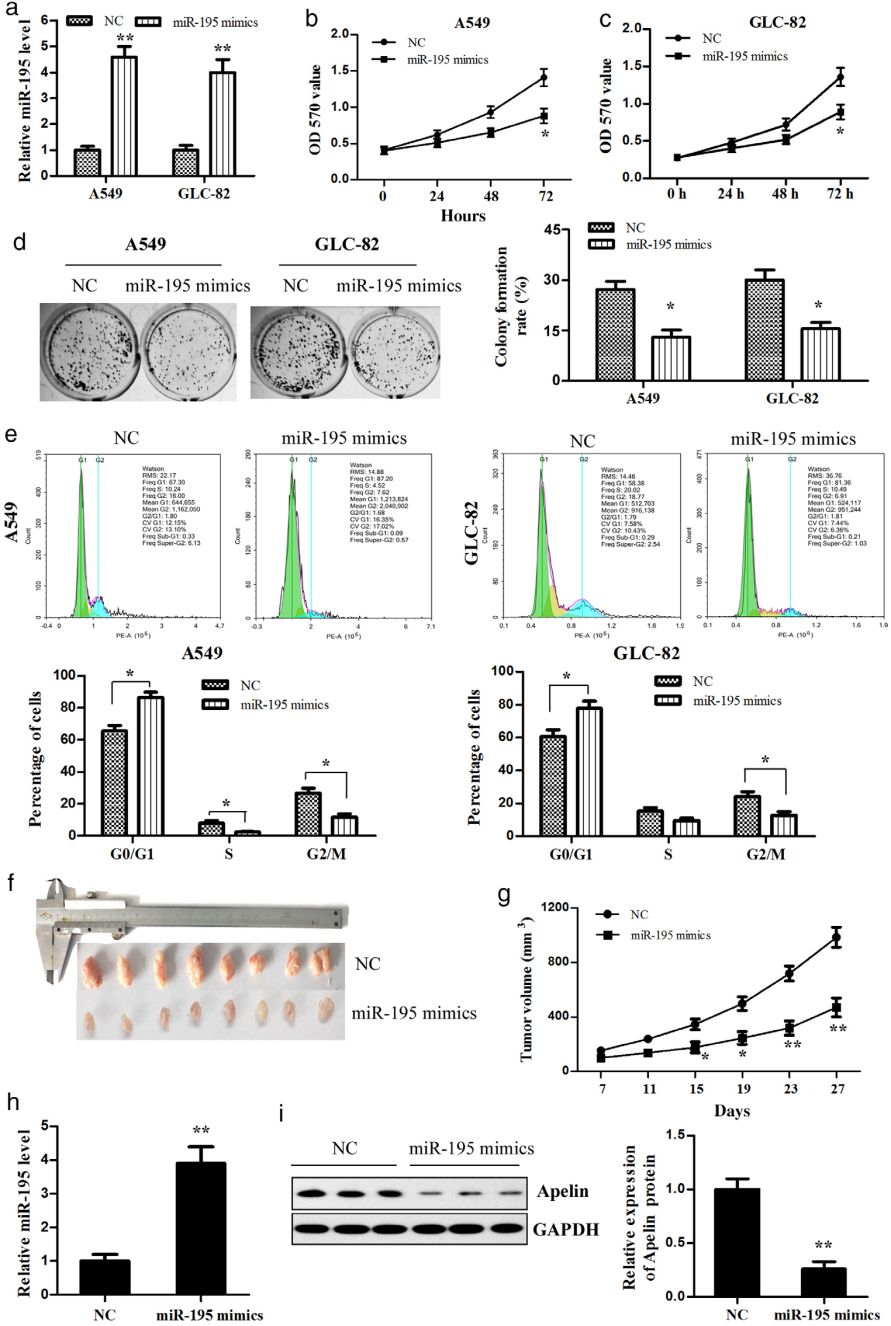
Overexpression of miR‐195 in lung adenocarcinoma cells, which inhibited their proliferation and growth in vitro and in vivo. (**a**) The miR‐195 mimics or negative control (NC) was transfected into A549 and GLC‐82 cells, respectively, and then miR‐195 expression was detected using quantitative reverse transcription (qRT) PCR. The cell viability of the (**b**) A549 and (**c**) GLC‐82 cells transfected with miR‐195 mimics was measured using methyl thiazolyl tetrazolium assay. (**d**) The colony formation of the transfected A549 and GLC‐82 cells was detected. (**e**) The effect of miR‐195 on the lung adenocarcinoma cell cycle was determined using flow cytometry. (**f**) A representative image of the xenograft tumors is shown (*n* = 8 per group). (**g**) The growth curve of xenograft tumors derived by miR‐195‐treated A549 cells was determined in vivo. (**h**) MiR‐195 and (**i**) apelin protein expression in tumor tissues was determined by qRT‐PCR and Western blotting, respectively. ^*^ *P* < 0.05, ^**^ *P* < 0.01. GAPDH, glyceraldehyde 3‐phosphate dehydrogenase.

To investigate whether increased levels of miR‐195 might affect lung adenocarcinoma cell proliferation, miR‐195 mimics or NC was transfected into A549 and GLC‐82 cells. QRT‐PCR showed that the miR‐195 mimics significantly increased the miR‐195 expression in both the A549 and GLC‐82 cells (Fig 4a). Our results demonstrate that miR‐195 overexpression inhibited the proliferation of A549 and GLC‐82 cells compared to NC (Fig 4b,c). Moreover, colony formation assay showed that miR‐195 overexpression decreased the colony formation ability of lung adenocarcinoma cells (Fig 4d). To ascertain the mechanisms by which miR‐195 inhibited the proliferation of lung adenocarcinoma cells, we detected the cell cycle distribution via flow cytometry. The G0/G1 phase fractions of the miR‐195‐A549 and miR‐195‐GLC‐82 cells increased by 1.31‐ and 1.25‐fold compared to that of the transfected cells with NC, respectively (Fig 4e). However, the G2/M phase fractions of the miR‐195‐A549 and miR‐195‐GLC‐82 cells decreased by 0.44‐ and 0.53‐fold, respectively. The percentages of cells in the S phase in the transfected cells with miR‐195 were lower than in the cells with NC. We also explored the potential involvement of miR‐195 in tumorigenesis through the A549 xenograft mouse model in vivo. The tumors were smaller in the miR‐195 mimics group compared to those in the NC group (Fig 4f). The mean volume of xenograft tumors was significantly smaller in the miR‐195 mimics group compared to the NC group (Fig 4g). Furthermore, we examined the miR‐195 and apelin expression levels in tumor tissues using qRT‐PCR and Western blot, respectively. The results revealed that miR‐195 expression was significantly upregulated, while apelin expression was downregulated in the miR‐195 mimics group compared to the NC group (Fig 4h,i). Therefore, we concluded that miR‐195 suppresses lung adenocarcinoma cell growth in vitro and in vivo.

### Overexpression of miR‐195 suppresses the migration and invasion of lung adenocarcinoma cells

**Figure 5:**
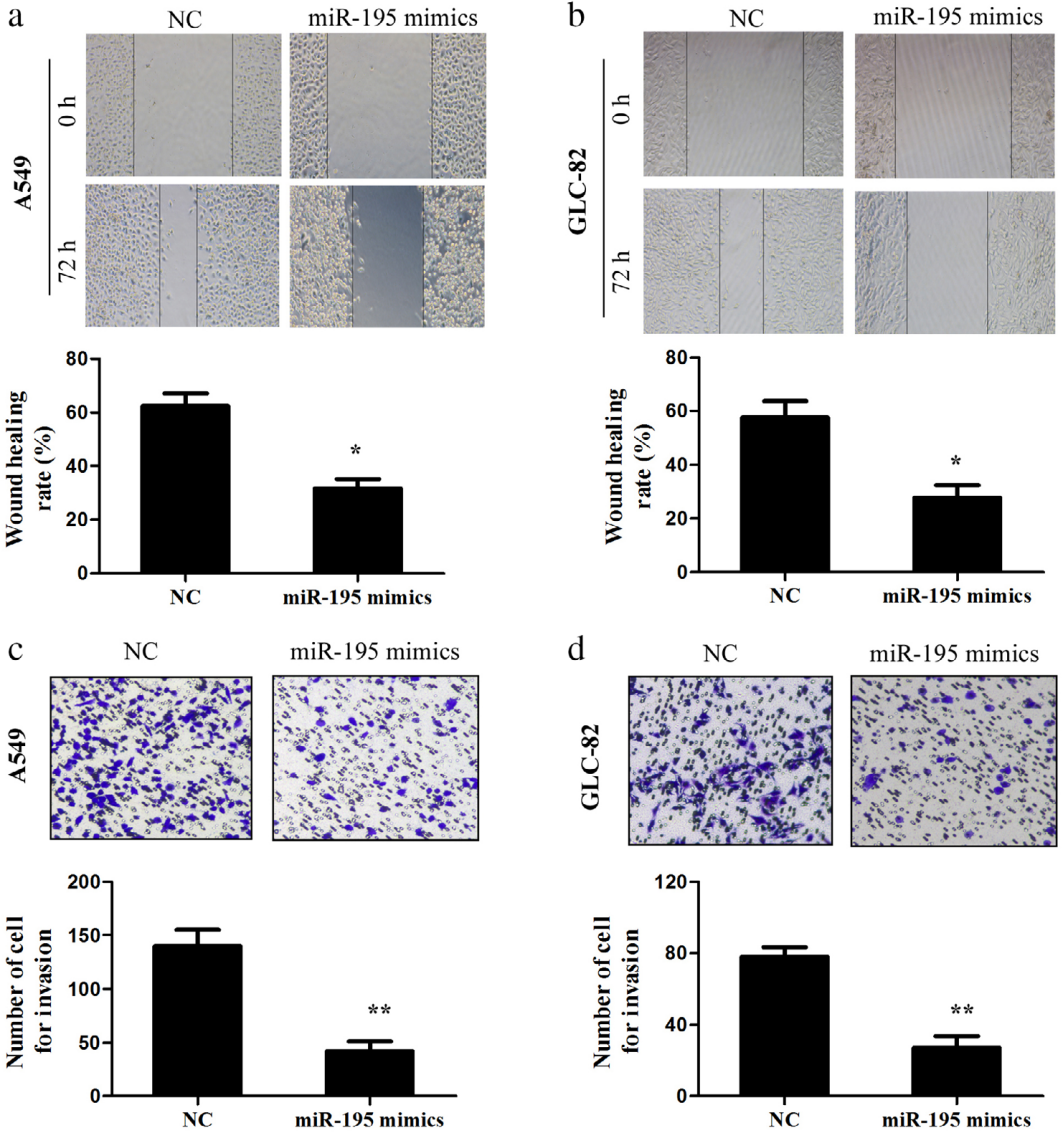
Overexpression of miR‐195 suppressed the migration and invasion of lung adenocarcinoma cells. (**a**,**b**) Cell migration was determined by wound‐healing assay in A549 and GLC‐82 cells after transfection with miR‐195 mimics or negative control (NC). (**c**,**d**) Transwell invasion assay was conducted in the A549 and GLC‐82 cells. ^*^ *P* < 0.05, ^**^ *P* < 0.01.

To confirm the results of previous studies that miR‐195 prohibits lung cancer cell migration and invasion, we performed wound‐healing and transwell assays. In the wound‐healing assay, increased miR‐195 expression significantly suppressed the cell migration ability of the A549 and GLC‐82 cells (Fig 5a,b). Moreover, the overexpression of miR‐195 induced a notable decrease in the invasion ability of the A549 and GLC‐82 cells compared to the cells transfected with NC (Fig 5c,d). Taken together, these data reveal that miR‐195 overexpression inhibits the migration and invasion of lung adenocarcinoma cells.

### MiR‐195 performs a tumor suppressor role in lung adenocarcinoma cells by downregulating apelin expression

**Figure 6:**
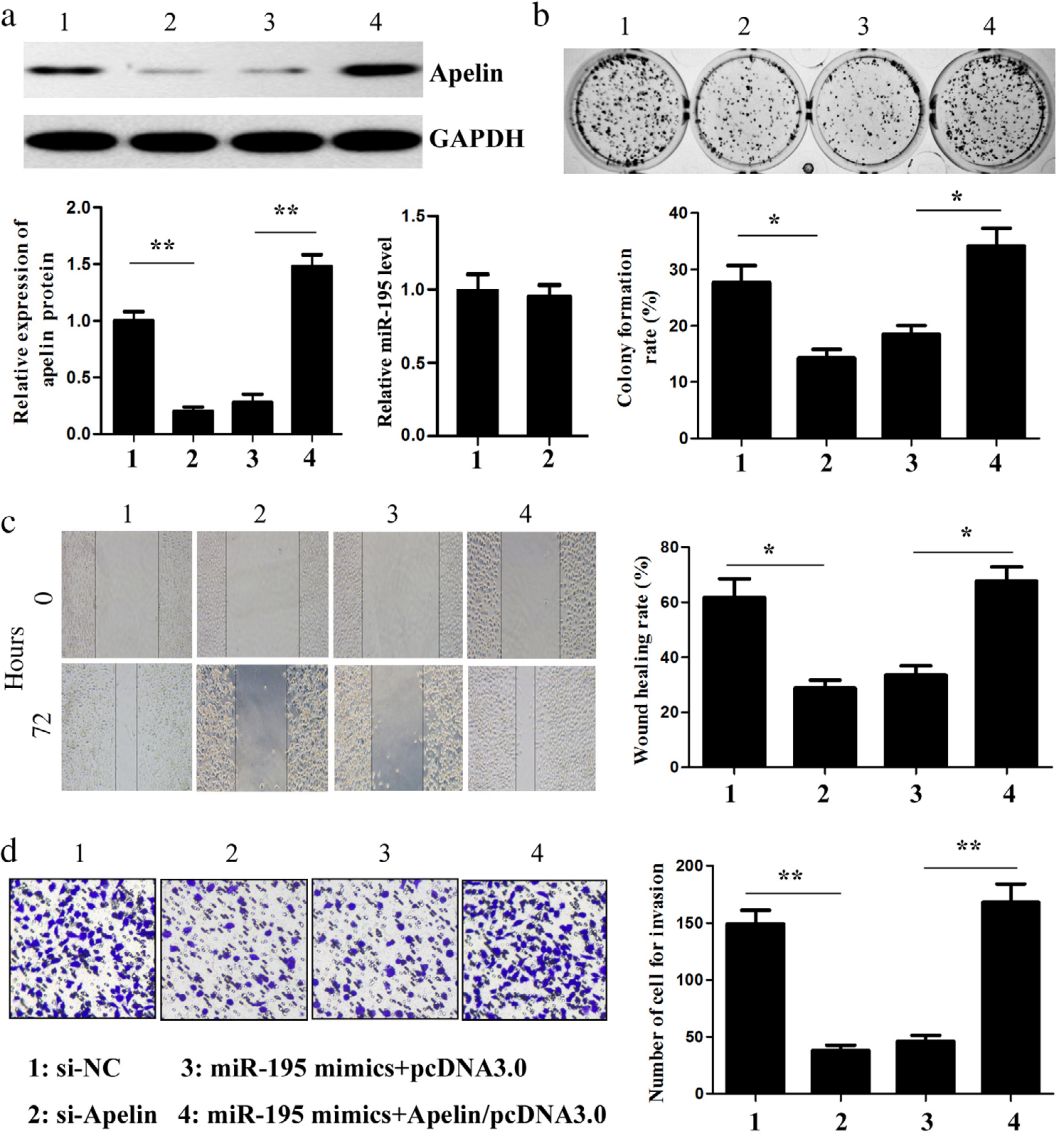
MiR‐195 downregulated apelin expression in lung adenocarcinoma cells. (**a**) The apelin protein and miR‐195 levels in the A549 cells cotransfected with small interfering‐apelin (si‐apelin) and cotransfected with miR‐195 mimics and apelin overexpression vector were measured by Western blotting and quantitative reverse transcription (qRT) PCR, respectively. (**b**) The colony formation of the transfected A549 cells was detected. The effects of miR‐195 on cell migration and invasion were determined using (**c**) wound‐healing and (**d**) transwell invasion assays, respectively (× 100). ^*^ *P* < 0.05, ^**^ *P* < 0.01. GAPDH, glyceraldehyde 3‐phosphate dehydrogenase.

In the present study, to further explore the function of apelin in the miR‐195‐mediated tumor suppressive behavior of lung adenocarcinoma cells, short hairpin RNA targeting apelin mRNA (si‐apelin) was used to specifically inhibit apelin expression, while the apelin/pcDNA3.0 vector was exploited to overexpress apelin in A549 cells. Si‐apelin significantly decreased the apelin protein level, while the apelin overexpression vector increased the protein level in the A549 cells (Fig 6a). Meanwhile, there was no significant difference in the level of miR‐195 between the si‐apelin and si‐NC groups. Furthermore, the inhibition of apelin expression observably reduced the proliferation, migration, and invasion of the A549 cells similar to the effects of the miR‐195 mimics, while the upregulation of apelin reversed the changes in A549 cell proliferation, migration, and invasion induced by the miR‐195 mimics (Fig 6b–d). These data suggest that miR‐195 suppresses lung adenocarcinoma cell malignancy by targeting apelin.

## Discussion

Lung adenocarcinoma accounts for the majority of NSCLC tumor histology. Although extensive studies have led to significant improvements in early detection and treatment options, the disease remains difficult to treat and many patients develop recurrent disease after surgery. The mean five‐year survival rate of patients with advanced disease after diagnosis is < 15%.^22^, ^23^ Therefore, many recent studies have focused on finding new prognostic biomarkers and pivotal molecular mechanisms associated with the development and metastasis of lung cancer.^24^, ^25^

Apelin is a regulatory peptide identified as an endogenous ligand of the apelin receptor APJ.^4^ Apelin and its receptor have been reported to stimulate endothelial growth in different in vitro and in vivo experimental systems,^26^, ^27^, ^28^ and some recent studies have shown that apelin expression is detected at a high levels in breast cancer,^8^ oral squamous cell carcinoma,^10^ and liver cancer.^11^ In the present study, we found that apelin is not only significantly upregulated in lung adenocarcinoma tissues compared to adjacent healthy lung tissues but also positively correlated with poor prognosis of patients with lung adenocarcinoma. These results suggest that high apelin expression may be closely related to the development and progression of lung adenocarcinoma.

The dysregulation of miRNA is implicated in the development and progression of various cancers, including lung adenocarcinoma.^29^, ^30^, ^31^ However, the function and mechanism of these dysregulated miRNAs in lung adenocarcinoma remain undefined. Recent research has shown that most miRNAs are known to regulate target mRNAs by binding to the 3′UTR of target genes in a posttranscriptional manner.^13^, ^14^ Therefore, establishing the interrelationship of miRNA and its target genes may increase the understanding of the molecular mechanism underlying cancer progression and provide potential therapeutic targets for the clinical treatment of cancers. Our study demonstrated that the levels of apelin mRNA and protein are both negatively regulated by miR‐195 in lung adenocarcinoma cells and the apelin expression level is inversely correlated with miR‐195 expression. In addition, luciferase reporter assay confirmed that *apelin* is a direct target gene of miR‐195 in lung adenocarcinoma cells. These data indicate that miR‐195 might play an important inhibitory role in lung adenocarcinoma cells by targeting apelin.

Accumulating evidence indicates that miR‐195 is associated with the biological features of cancers, such as oncogenesis, development, and metastasis.^32^, ^33^, ^34^, ^35^, ^36^ Recent studies have shown that miR‐195 is frequently downregulated or silenced in many types of cancers, including osteosarcoma,^32^ breast cancer,^33^ colon cancer,^34^ prostate cancer,^35^ and glioma.^36^ In these different cancers, miR‐195 usually acts as a tumor‐suppressive factor. The current study found that miR‐195 expression was significantly downregulated in human lung adenocarcinoma tissues and cell lines, and the expression level of miR‐195 was positively correlated with the prognosis of lung adenocarcinoma patients. Our functional studies revealed that the overexpression of miR‐195 could suppress cell proliferation, colony formation, migration, and invasion of lung adenocarcinoma in vitro. Hanahan *et al.* reported that antigrowth signals can block cell proliferation via two distinct mechanisms: cells may be forced out of the active proliferative cycle into the quiescent (G0) state, or cells may be induced to enter post‐mitotic states and lose the potential to differentiate.^37^ Our study found that the overexpression of miR‐195 in lung adenocarcinoma cells may result in the activation of antigrowth signals, inducing cell cycle arrest at the G0/G1 phase and disturbance to the G1 to S phase transition. Furthermore, the results revealed that tumor growth in the nude mice was obviously inhibited by the increased miR‐195 expression in the A549 cells. Briefly, these data suggest that miR‐195 may serve as a negative regulator in the tumorigenesis and development of lung adenocarcinoma.

In the present analysis of clinical human lung adenocarcinoma tissues, the apelin mRNA level was negatively correlated with the expression level of miR‐195. In addition, functional research also showed that apelin was positively correlated with cell proliferation, migration, and invasion of lung adenocarcinoma. Collectively, high miR‐195 and low apelin work synergistically, suppressing tumor growth and improving the survival outcome of lung adenocarcinoma patients. Taken together, these results confirm that apelin is a functional target of miR‐195 in lung adenocarcinoma cells and validates the function of miR‐195‐apelin interaction in the progression of lung adenocarcinoma. Further investigation is required to elucidate the role of molecules that are downstream of apelin in regulating cell proliferation, migration, and invasion of lung adenocarcinoma.

In conclusion, this study identified miR‐195 as a tumor‐suppressive factor in human lung adenocarcinoma by directly targeting apelin. These findings suggest that miR‐195 and its target, apelin, may be novel target candidates for lung adenocarcinoma therapeutics in the future.

## Disclosure

No authors report any conflict of interest.
